# Does parental monitoring moderate the relationship between bullying and adolescent nonsuicidal self-injury and suicidal behavior? A community-based self-report study of adolescents in Germany

**DOI:** 10.1186/s12889-015-1940-x

**Published:** 2015-06-24

**Authors:** Vanessa Jantzer, Johann Haffner, Peter Parzer, Franz Resch, Michael Kaess

**Affiliations:** Department of Child and Adolescent Psychiatry, Center for Psychosocial Medicine, University of Heidelberg, Blumenstraße 8, Heidelberg, 69115 Germany

**Keywords:** Bullying, Suicidal behavior, Nonsuicidal self-injury, Parenting, Adolescent, Schools

## Abstract

**Background:**

Being a victim of bullying in school is clearly linked to various social, emotional, and behavioral problems including self-harm behavior. However, it is not known whether even occasional victimization has similar negative consequences and whether protective factors such as social support may prevent those harmful developments. The present study therefore focuses on the nonsuicidal self-injury (NSSI) and suicidal behavior (SB) in victims of bullying and the potentially moderating effect of parental monitoring.

**Methods:**

In all, a cross-sectional sample of 647 adolescents (mean age 12.8 years) were surveyed concerning bullying experiences, NSSI and SB, and parental monitoring.

**Results:**

A total of 14.4 % of respondents reported being a victim of frequent bullying in the past few months (with verbal and social bullying playing the most important role), which increased the risks of both NSSI (OR = 11.75) and SB (OR = 6.08). This relationship could also be shown for occasional victims of bullying (35.6 %), although to a lesser extent. Parental monitoring had a significant protective effect on SB in victims of occasional bullying. However, parental monitoring did not show any protective effect in victims of repetitive bullying.

**Conclusions:**

Victims of bullying show a substantial risk for engaging in self-harm behavior. Therefore, the dissemination of anti-bullying programs in schools would probably also prevent such disorders. Parental participation in school-based prevention may increase its effect; this also matches the results of the present study, showing that parental monitoring may be able to buffer the negative effects of bullying victimization, at least to a certain degree.

## Background

School bullying is a major social problem affecting children and adolescents in all parts of the world. Bullying is defined as repeated negative actions over a longer period. These negative actions can be performed by a single person or group and carried out in direct (i.e., physical or verbal) or indirect (i.e., social) form. The key criteria of bullying are the harmful intent of the perpetrator as well as an existing imbalance of power, which makes it difficult for the victim to defend himself [1]. A relatively new form of bullying is so-called cyber bullying, i.e., bullying via mobile phone or Internet. Most representative international studies indicate that 20 to 30 % of all students are affected by bullying, as victims, perpetrators, or bully/victims [1]. However, the large-scale study “Health Behaviour in School-Aged Children” (HBSC), which collected data from more than 200,000 adolescents in 40 European countries, showed variations ranging from 7 to 40 %, indicating considerable cultural differences [2]. The distress and suffering caused by school-based bullying is enormous, sometimes having long-term consequences into adulthood [3]. Being a victim of bullying is linked to various academic, social, emotional, and behavioral problems [4].

Most research so far has focused on overall experiences of bullying. However, bullying represents a wide range of experiences and can be differentiated into various subtypes, such as verbal, social, physical, and cyber bullying [5], or can also be categorized by the frequency of these experiences. A phenomenon of particular interest in the past years has been cyber bullying. However, it has been investigated less comprehensively due to its relative novelty [6]. In general, it is still not known whether certain types of bullying are particularly linked to an increased risk for certain disorders or whether the consequences of occasional or subliminal victimization are similar to those for victimization on a regular basis. For example, it could be hypothesized that some forms might cause severer mental health problems than others: social bullying because social status is especially related with psychological well-being during adolescence; or cyber bullying, with its special characteristics of lack of control, ubiquity and low inhibition threshold.

Previous research has identified bullying as a risk factor for adolescent self-harm behavior [3, 7–9]. These results may be in line with findings that determined bullying as a factor in the development of severe emotional and behavioral problems [4, 10] or borderline personality pathology [11]. Nevertheless, drawing causal conclusions are not justified to date, and even longitudinal designs cannot rule out the possibility of endogeneity meaning that those with severe emotional problems are more likely to be bullied (see limitations below).

Adolescent self-harm behavior includes both nonsuicidal self-injury (NSSI) and suicidal behavior (SB). Both are common among adolescents and therefore have gained increasing public and scientific attention during the past decade. The mean prevalence of NSSI was recently reported to be 18 % in nonclinical adolescent populations [12] while the mean proportion of adolescents reporting suicidal thoughts and suicide attempts in the previous year were 19.3 and 6.4 %, respectively [13]. The importance of NSSI and SB has just been highlighted now that ‘nonsuicidal self-injury disorder’ and ‘suicidal behavior disorder’ have been included in section 3 of the new Diagnostic and Statistical Manual for Mental Disorders (DSM-5) [14], where NSSI is described as intentional self-inflicted damage to the surface of an individual’s body without conscious suicidal intent. This typically involves cutting or carving the skin [15], but also other forms like self-biting, hitting self on purpose, or burning skin [16].

While there is a large body of evidence concerning the risk factors for NSSI and SB, both the protective factors and the interaction between risk and protective factors still represent largely unanswered questions. With regards to the association between bullying and adolescent self-harm, the remaining question is: Why do many victims of bullying show resilience and not develop self-harm behaviors? Potential protective factors in the context of bullying may include certain school characteristics (student relationships with teachers, school atmosphere, etc.), individual characteristics (e.g., personality factors), and social protection through parents or friends. Studies on the protective effect of social support have produced mixed results so far. Sainio et al. [17] showed that victims who experienced support from peers were less depressed and anxious and had more self-confidence than victims without any social support. The results from Kendrick et al. [18] confirmed the so-called friendship protection hypothesis, but only for boys. However, Rothon et al. [19] emphasized that the support from friends and parents acts as a buffer on the school performance of the bullying victims, but has no influence on the development of depression. Yet another study, however, found that parental support moderated the relationship between victimization and internalizing distress from bullying in female adolescents only [20].

To the best of our knowledge, there are no data concerning protective factors moderating the relationship between different experiences of bullying and the development of NSSI and SB. Parental factors may be of particular importance as adolescent self-harm behaviors have previously been linked to parent–child relationship problems [15], and a recent study on a large European sample of adolescents identified a strong association between adolescent self-injury and lack of parental monitoring over and above emotional and behavioral problems [21]. According to Lowe and Dotterer [22], parental monitoring is a parenting practice that is defined as parents’ knowledge of their adolescents’ school and social activities and whereabouts. While diminished parental monitoring may be a risk factor for NSSI and SB, it can be hypothesized that adequate parental monitoring may protect victims of bullying from engaging in self-harm behaviors because parents may identify the bullying, provide emotional support to their children, engage with school staff and other parents to stop the bullying of their offspring, and seek professional help for their victimized children.

The present study aimed 1) to assess the relationship between bullying and NSSI/SB in a community-based sample of adolescents, also considering frequency and type of bullying; and 2) to investigate parental monitoring as a potential protective moderator of this relationship.

## Methods

### Recruitment and procedure

Data were collected within the *“Projekt Weichensteller”*, an academic support project for school social work in the city of Heidelberg, Germany. Before commencing the study, the ethics committee of the University of Heidelberg and the respective school authorities approved the study and written and informed consent was obtained from all children and their respective caregivers. Students were assessed from April to July 2012 using self-report questionnaires. The assessments took place during regular class times; the duration was a maximum of 45 min. All participants took part in a raffle as an incentive for participation.

### Sample

The data presented are student data (grades 5, 7, and 9). In all, 647 students (50.7 % females) between 9 and 18 years of age participated in the study. The mean age of the participants was 12.8 years (SD = 1.95); 267 students (41.3 %) were in grade 5 (mean age = 10.9 years), 195 (30.1 %) were in grade 7 (mean age = 13.1 years), and 185 (28.6 %) were in grade 9 (mean age = 15.3 years).

### Measures

Experiences of bullying were measured using the Revised Olweus Bully/ Victim Questionnaire (BVQ-R), subscale victimization. The BVQ-R is a widely used instrument with a clear definition of bullying. Students are asked how frequently they have engaged in different bullying behaviors in the past few months (“It hasn’t happened to me in the past couple of months”, “Only once or twice”, “2 or 3 times a month”, “About once a week”, or “Several times a week”). The commonly used cut-off for bullying is “2 or 3 times a month”, which we defined as repetitive bullying, while occasional bullying was defined as “Only once or twice”. The subscale ‘victimization’ consists of ten items which can be assigned to the five different types of bullying (physical, verbal, social, cyber, and other).

NSSI was assessed using a single-item which clearly distinguished NSSI from SB by its intent (“… without the intention to kill you”). NSSI was rated by frequency within the last 12 months and was categorized into occasional self-harm (“1–4 times”) and repetitive self-harm (“At least 5 times”).

SB was recorded using the Paykel Suicide Scale (PSS) [23], which distinguishes six degrees of severity within the last 12 months. SB was categorized into longing for death, suicidal thoughts or plans (“thoughts of taking life” or “seriously considered taking life”), and suicide attempts.

A seven-item scale was developed to assess parental monitoring (“When I am not at home, my parents know where I am and who I am with”, “We have clear rules in the family”, “We eat at least one meal together as a family each day”, “The subject is raised and there are consequences if I break rules”, “My parents keep a check on what I watch on television”, “In the afternoon/evening I am often alone at home”, and “My parents keep a daily check on my homework”), to which adolescents responded on a three-point scale (“Not true”, “Somewhat true”, or “Certainly true”).

### Statistical analyses

Descriptive statistics were calculated for all bullying groups. Group comparisons between categorical variables were analyzed using chi-squared tests. Using logistic regression for ordered categories (proportional odds model), the odds ratios indicating the strength of the relationship between bullying, NSSI, and SB were determined. In a second step, parental monitoring was included as a moderator variable. In a third step we explored the possibility, that different types of bullying have different effects on NSSI and SB using logistic regressions for ordered categories of NSSI and SB on five types of bullying (physical, social, verbal, cyber, and other). A subsequent stepwise reduction of the regression models were conducted in order to minimize the Bayes Information Criterion (BIC) [24]. Effect sizes of the regression models are described as proportion of explained information (McFadden’s Pseudo R^2^). For better interpretability, the 95 % confidence intervals and p-values (Wald test) are indicated in each case. Data were analyzed using Stata 13 [25].

## Results

Descriptive analyses showed that 93 students (14.4 %) reported having been a victim of repetitive bullying in the last few months, with verbal (9.7 %) and social bullying (8.0 %) being the most frequent. Furthermore, 230 students (35.6 %) reported having been occasionally victimized in the last few months, with social (23.5 %), physical (20.9 %) and verbal bullying (19.7 %) being the most frequent. Table 1 includes the frequency of all bullying categories assessed. Spearman’s rank correlations between these different types of bullying were calculated. The obtained values ranged between .21 (verbal and cyber bullying) and .46 (verbal and social bullying).

**Table 1:** Self-reported frequency of victimization (*N* = 647), differentiated for type of bullying

	Frequency of bullying					
	None		Occasional		Repetitive	
	N	%	N	%	N	%
Physical bullying	486	75.0	135	20.9	26	4.0
Verbal bullying	457	70.6	127	19.6	63	9.7
Social bullying	443	68.5	152	23.5	52	8.0
Cyber bullying	617	95.4	19	2.9	11	1.7
Other type of bullying	602	93.0	33	5.1	12	1.9
Bullying total	324	50.1	230	35.6	93	14.4

Occasional bullying was defined as “Only once or twice in the past couple of months”, repetitive bullying means at least “2 or 3 times a month”

There were no significant gender differences in the frequency of bullying (χ^2^(2) = 0.5, *p* = .78); however, bullying frequency differed between grades (χ^2^(4) = 10.82, *p* = .029). An inspection of the frequencies showed that occasional bullying decreases substantially between grade 7 and grade 9, while repetitive bullying does not change from grade 5 to grade 9.

A total of 48 participants (7.6 %) indicated they had occasionally and 23 (3.6 %) repetitively engaged in NSSI in the past 12 months. Moreover, 49 students (7.7 %) reported longing to die, 86 (13.4 %) reported suicidal thoughts or plans, and 17 (2.7 %) reported suicide attempts in the past 12 months.

As expected, both NSSI (χ^2^(2) = 17.09, *p* < .001) and SB (χ^2^(3) = 26.72, *p* < .001) were more common in girls. Additionally, a clear effect of age could be shown. While grade 7 and 9 students did not differ with regard to their self-harm behavior, grade 5 students reported significantly less NSSI (χ^2^(4) = 21.87, *p* < .001) and SB (χ^2^(6) = 30.32, *p* < .001).

The described self-harm behavior differentiated for the adolescents’ victimization status is presented in Table 2.

**Table 2:** Self-reported prevalences of suicidal behavior (*N* = 640) and nonsuicidal self-injury (*N* = 636) differentiated for victimization status

	Frequency of bullying							
	None		Occasional		Repetitive		Total	
	N	%	N	%	N	%	N	%
Suicidal behavior								
None	279	86.7	160	70.8	49	53.3	488	76.3
Longing for death	15	4.7	19	8.4	15	16.3	49	7.7
Suicidal thoughts/plans	23	7.1	39	17.3	24	26.1	86	13.4
Suicide attempts	5	1.6	8	3.5	4	4.4	17	2.7
Nonsuicidal self-injury								
Never	306	96.2	193	85.4	66	71.7	565	88.8
1-4 times	8	2.5	25	11.1	15	16.3	48	7.6
5+ times	4	1.3	8	3.5	11	12.0	23	3.6

For the estimation of the odds ratios between bullying and NSSI and SB, we included grade as a covariate, since grade was related to both bullying as well as NSSI and SB. Regression analyses clearly showed that being a victim of repetitive bullying was significantly associated with both NSSI and SB. With regards to repetitive bullying, we found an odds ratio (OR) of 6.08 (95 %CI = 3.62 – 10.21, *p* < .001) for SB and 11.75 (95 % CI = 5.54 – 24.94; *p* < .001) for NSSI. Significantly increased, but smaller ORs could also be shown for occasional victims of bullying at 2.89 (95 %CI = 1.86 – 4.50; *p* < .001) for SB and 4.74 (95 %CI = 2.36 – 9.54; *p* < .001) for NSSI. The increase of the explained information for the prediction of SB was R^2^ = 0.053 and for the prediction of NSSI was R^2^ = 0.093. Interactional effects of bullying with gender and bullying with grade were tested in the regression models for both NSSI and SB but revealed no significant effects.

The different types of bullying and their relationship with NSSI and SB resulted in a quite complex pattern (see Tables 3 and 4). While social bullying seemed to be a triggering factor for both NSSI and SB, cyber bullying showed an especially strong relationship with repetitive NSSI.

**Table 3:** Results of ordered logistic regressions with suicidal behavior as dependent variable and types of bullying as independent variables

	Frequency of bullying					
	Occasional			Repetitive		
	OR	*p*	95 % CI	OR	*p*	95 % CI
Full model						
Physical bullying	1.45	.119	0.91-2.32	1.34	.556	0.51-3.51
Verbal bullying	1.71	.032	1.05-2.79	1.68	.167	0.81-3.50
Social bullying	2.43	<.001	1.55-3.80	3.29	.001	1.59-6.82
Cyber bullying	1.44	.456	0.55-3.81	1.07	.922	0.29-3.87
Other bullying	0.51	.132	0.21-1.23	0.99	.992	0.28-3.55
Reduced model with minimum BIC						
Social bullying	3.02	<.001	2.00-4.57	5.55	<.001	3.16-9.76

Explained information for the full model R^2^ = 0.06, explained information for the reduced model R^2^ = 0.05. The correlations of the parameter estimates are between -.37 and .38

**Table 4:** Results of ordered logistic regressions with nonsuicidal self-injury as dependent variable and types of bullying as independent variables

	Frequency of bullying					
	Occasional			Repetitive		
	OR	*p*	95 % CI	OR	*p*	95 % CI
Full model						
Physical bullying	1.11	.754	0.58-2.11	1.67	.422	0.48-5.81
Verbal bullying	1.97	.042	1.02-3.79	1.27	.646	0.46-3.47
Social bullying	3.05	<.001	1.63-5.70	4.91	.001	1.93-12.5
Cyber bullying	2.05	.193	0.70-6.04	9.08	.002	2.22-37.1
Other bullying	0.84	.745	0.29-2.45	0.41	.305	0.08-2.24
Reduced model with minimum BIC						
Social bullying	3.60	<.001	2.00-6.47	5.84	<.001	2.68-12.7
Cyber bullying	2.45	.084	0.89-6.80	10.0	<.001	2.83-35.3

Explained information for the full model R^2^ = 0.12, explained information for the reduced model R^2^ = 0.11. The correlations of the parameter estimates are between -.40 and .46

**Fig. 1:**
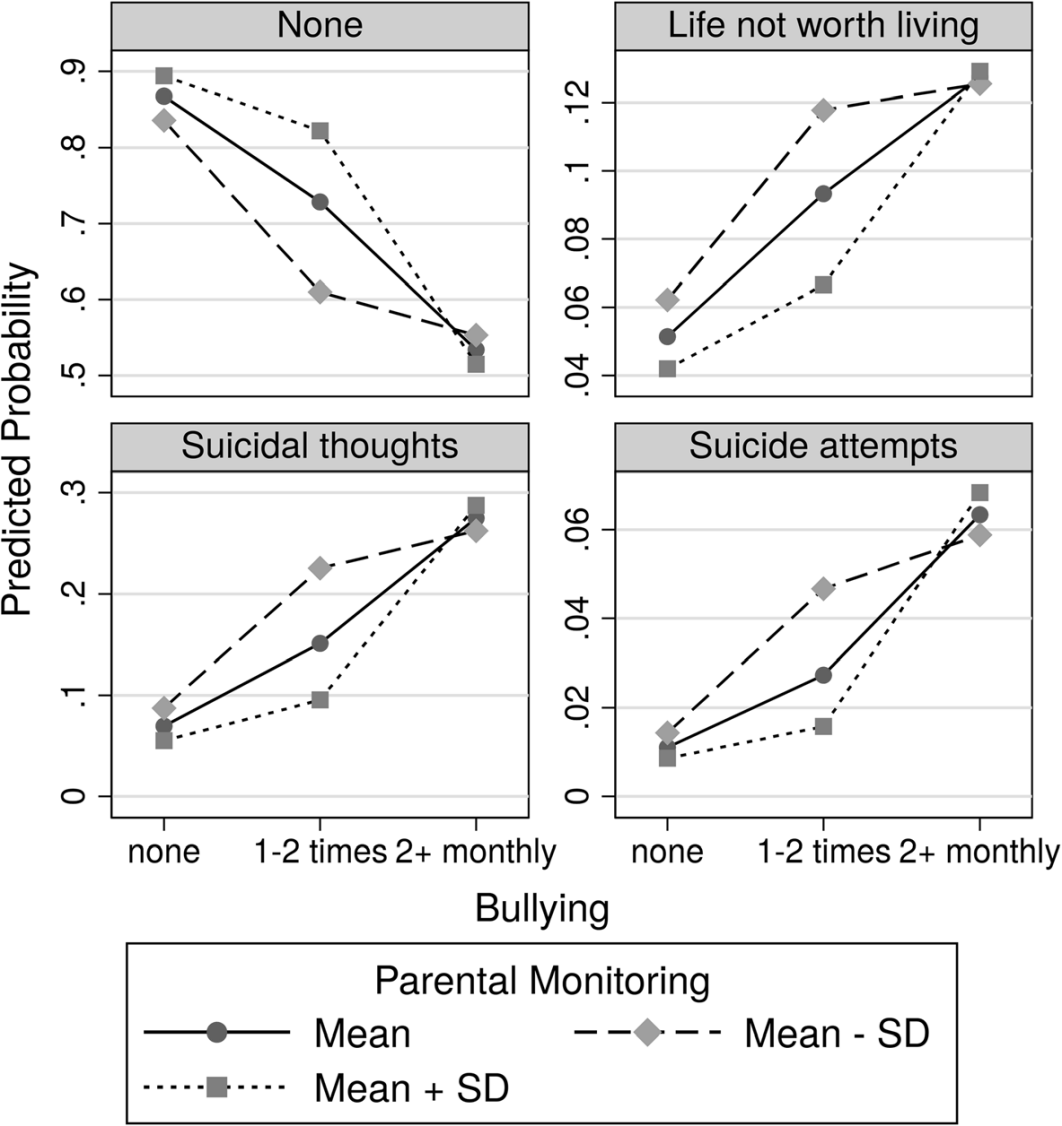
Marginal predicted probabilities for the different categories of suicidal behavior as a function of the frequency of bullying and for low (mean minus 1 SD), mean and high (mean plus 1 SD) parental monitoring

A significant interaction between bullying and parental monitoring could be found for SB (χ^2^(2) = 7.41, *p* = .025), but not for NSSI (χ^2^(2) = 1.79, *p* = .409). Looking at the relationship between bullying and SB in detail, parental monitoring had a protective effect on SB for adolescents who had experienced occasional bullying (OR = 0.57, 95 %CI = 0.41 – 0.79, *p* < .001). Parental monitoring, however, did not show a protective effect for adolescents who had not been victims of bullying at all (OR = 0.77, 95 %CI = 0.55 – 1.09, *p* = .142) and for victims of repetitive bullying (OR = 1.08, 95 %CI = 0.77 – 1.53, *p* = .648). Figure 1 illustrates this complex relationship.

For example, the graph “Suicide attempts” shows the probabilities for suicide attempts as predicted by the logistic regression model. For subjects without bullying experience, the probabilities are low (between *p* = .009 and *p* = .014) and the difference between the probabilities for subjects with low or high parental monitoring is small. For victims of occasional bullying, the probabilities of suicide attempts increase, but this increase depends on parental monitoring. With low parental monitoring, the probability increases more than three times from *p* = .014 to *p* = .047, while with high parental monitoring, the probability increases less than two times from *p* = .009 to *p* = .016. For victims of repetitive bullying, the probabilities for suicide attempts are between *p* = .058 and *p* = 0.068 with again a small difference between subjects with low or high parental monitoring. The same applies to the other graphs representing different forms of SB.

## Discussion

In our study the prevalence of bullying is comparable to that reported in representative international studies [26, 27], which supports the generalizability of the results. The initially low prevalence of NSSI and SB can be explained by the rare occurrence of these behaviors in grade 5. Previous data indicate that self-harm behaviors usually first occur between age 14 and 18 [28]. Comparable population-based studies have mainly included adolescents 14 years and older, which may most likely explain the overall higher prevalence.

The present findings clearly show that victims of frequent bullying at school show a substantial risk for engaging in both NSSI and SB. This finding is in line with the previous literature [3, 7–9]. However, our study brings up some important new aspects. First of all, the described relationship already seems to be present for occasional victims of bullying, those who have been affected only once or twice in the last few months and actually do not meet the common cut-off for bullying. Not surprisingly, this effect could be shown to a lesser extent (dose–response relationship). Another dose–response relationship has been reported previously: victims of multiple types of bullying are more troubled than victims of only one type [29, 30]. However, previous studies have not related this to the frequency of bullying. As a consequence, children who have been victimized occasionally usually do not apply as victims of bullying, even if they are also at risk for adverse mental health outcomes, as can be shown by our data.

Our data showed that social bullying had a particularly serious impact because of its relationship with both NSSI and SB. Considering adolescence as the developmental phase in which building relationships with peers, finding social roles, and detaching oneself from the family are the most important tasks, this strong relationship seems to be reasonable. Moreover, the results may fit to the observation that adolescent self-harm often occurs in social contexts (associated with feelings of rejection or loneliness) and may be used to regulate affect (self-harm as a strategy for coping with emotional distress) or interpersonal influence (self-harm to influence people in the self-injurer’s environment or as a cry for help) [31].

Olweus points out that cyber bullying, despite the current media attention, seems to be a relatively rare phenomenon [32]. Two large samples in the U.S. and Norway showed that the prevalence of cyber bullying was around 3 to 5 %, while verbal bullying was about four times as common with 11 to 18 %. Contrary to the general notion, cyber bullying did not increase during the study period from 2006 to 2010 and has created virtually no new victims and perpetrators, as the overlap with victims and perpetrators of traditional bullying types was approximately 90 %. Nonetheless, although it was the most infrequent type of bullying in our sample, cyber bullying showed an especially strong association with repetitive NSSI. According to Whitlock et al. [33], the Internet provides information and connects communities of individuals with shared interests or behaviors and therefore seems to be especially attractive for adolescents practicing repetitive NSSI. Indeed, sharing experiences in virtual communities makes young people more vulnerable to cyber bullying. In this case, the cyber bullying experience would not be a reason for NSSI, but a consequence of it.

While stronger parental monitoring significantly reduced the risk of SB for occasional victims, it did not make a difference for frequent victims and had no significant influence on the development of NSSI. These data somehow contradict a recently published study showing that both parenting and bullying prospectively predicted adolescent depressive symptoms but did not interact in the prediction of depressive thoughts and symptoms [34]. However, other data point towards an interaction of family environment and the relationship between bullying and self-harm behavior: Compared with bullied children who did not engage in self-harm, bullied children who did engage in self-harm were distinguished by a family history of attempted/completed suicide and a history of physical maltreatment by an adult [8]. In addition, there are data showing that positive parenting behavior was generally protective against peer victimization [35]. Our data suggest that parenting may be able to buffer bullying victimization but only to a certain degree. In addition, this buffering effect was only found for SB, potentially implying that the parent–child relationship may prevent adolescents from wanting to die, but not from developing mental health problems or self-injurious and risk-taking behavior. In general, the assessment of parental monitoring did not assess parents’ reactions to their child’s bullying experiences but rather a general trait of parenting behavior. Future research might in depth investigate how parents could deal with their offspring bullying experiences and might also include assessment of parents’ personality and psychopathology.

### Limitations

First, the cross-sectional nature of the study limits our ability to draw directional conclusions from our data. It is possible that both NSSI and SB are a cause rather than an outcome of bullying. Alternately, it is also possible that these problem behaviors are caused by a third underlying variable (e.g., childhood adversity or personality pathology). Therefore, further waves of this data set will be integrated to a prospective study. Even more important, intervention studies aiming to reduce bullying are needed to ultimately clarify the directional and causal relationship between bullying and adolescent self-harm behaviors. Such a study will be conducted by our study group on behalf of the Baden-Württemberg foundation, evaluating the Olweus Bullying Prevention Program concerning its effect on self-reported bullying prevalence but also on self-reported psychological health. For this purpose, the program will be implemented and academically supported in several pilot schools in Germany from 2015 until 2017.

Second, our data are derived from students’ self-reports only, and other sources of information, such as clinical interviews for the assessment of NSSI and SB should be considered in future studies. For measuring bullying, self-report surely is the most valid method. Bullying might happen quite subtle, so that others like teachers or parents often do not know what is going on, especially for social bullying. Therefore the correlations of self-report and measures involving other sources of information are often quite small [36]. Although the relationship between victimization and a variety of psychosocial disorders reflected in larger effect sizes in self-reports, effects are still present after the inclusion of multiple sources, as the meta-analysis of Hawker and Boulton showed [4].

Finally, the collected observations might be correlated because multiple subjects were drawn from the same schools and grades. Unfortunately, these correlations cannot be taken into account via multi-level regressions because we do not have reliable information about individual grades and schools. In many cases only grade level and type of school were documented.

### Implications

The serious consequences of bullying have generated a considerable amount of attention from the media, the public and healthcare practitioners. Nevertheless, in most countries there is a lack of clear policies and guidelines to counter the problem of bullying in school.

The manifold adverse effects of bullying underline the fact that bullying is an important public health concern, and not only an educational issue. The school setting, however, where bullying usually takes place, seems to be the ideal place to introduce prevention efforts. Primary prevention programs may protect against the stigmatization of affected individuals and offer the advantage that bystanders of bullying can also be reached and possible courses of actions can be shown to them. Furthermore, occasional victims of bullying, who are already under higher strain, can be reached by primary prevention as well. The current preventive focus on cyber bullying steers resources into the wrong direction, as traditional bullying, especially the social and verbal type, seems to be a much larger problem. Schools should therefore focus their prevention strategies on traditional bullying. If this can be reduced, cyber bullying will probably decline as well [32].

A recent meta-analysis by Ttofi and Farrington, which included 41 studies about different bullying prevention programs, revealed that (1) triggering factors of bullying can be positively influenced in the school context [37], and that (2) school-based prevention is effective in total, with a significant decrease in bullying of about 20 % across all programs. Moreover, investigation of effective intervention components revealed that parent meetings were more effective, which also matches the results of the present study. While purely parent programs would certainly not be sufficient for successfully preventing bullying per se, strengthening parental monitoring, protection, and care may well reduce the pathogenic effect of bullying victimization to a certain extent.

## Conclusions

Bullying is a potential risk factor for serious psychological problems, including NSSI and SB. Bullying prevention in schools could thus possibly also prevent adolescent self-harm behavior, an issue which needs to be addressed in intervention studies. Since parenting may potentially alleviate the pathogenic effects of bullying, parents should be involved in bullying prevention programs.
